# Peripartal management of dichorial twin pregnancy in a bicornuate bicollis uterus: a case report and review of the literature

**DOI:** 10.1186/s13256-024-04506-2

**Published:** 2024-04-21

**Authors:** Melanie Schubert, Anastasia Tihon, Kristin Andresen, Zino Ruchay, André Farrokh, Nicolai Maass, Philipp Elischer, Ann Carolin Longardt, Karolin Tesch, Annett Lebenatus, Magret Krüger, Ibrahim Alkatout

**Affiliations:** 1 Department of Obstetrics and Gynecology, University Hospital of Schleswig Holstein, Campus Kiel, 24105 Kiel, Germany; 2 Department of Obstetrics and Gynecology, University Hospital of Würzburg, 97080 Würzburg, Germany; 3 Department for Pediatrics and Adolescent Medicine I, Neonatology, University Hospital of Schleswig Holstein, Campus Kiel, 24105 Kiel, Germany; 4 Department of Radiology and Neuroradiology, University Hospital Schleswig-Holstein, Campus Kiel, 24105 Kiel, Germany; 5 Center for Operative Gynecology, Park Clinic, 24116 Kiel, Germany

**Keywords:** Dichorial twin pregnancy, Bicornuate unicollis uterus, Bicornuate bicollis uterus, Uterine malformations, Preterm birth

## Abstract

**Introduction:**

The management of a pregnancy in a bicornuate uterus is particularly challenging. A bicornuate uterus is a rare occurrence and a twin pregnancy in a bicornuate uterus even more rare. These pregnancies call for intensive diagnostic investigation and interdisciplinary care.

**Case presentation:**

We report on a 27-year-old European woman patient (gravida I, para 0) with a simultaneous pregnancy in each cavity of a bicornuate bicollis uterus after embryo transfer. The condition was confirmed by hysteroscopy and laparoscopy. Several unsuccessful *in vitro* fertilization (IVF) attempts had been performed earlier before embryo transfer in each cornus. After a physiological course of pregnancy with differential screening at 12 + 6 weeks and 22 + 0 weeks of gestation, the patient presented with therapy-resistant contractions at 27 + 2 weeks. This culminated in the uncomplicated spontaneous delivery of the leading fetus and delayed spontaneous delivery of the second fetus.

**Discussion:**

Only 16 cases of twin pregnancy in a bicornuate unicollis uterus have been reported worldwide and only 6 in a bicornuate bicollis uterus. The principal risks in such pregnancies are preterm labor, intrauterine growth restriction, malpresentation and preeclampsia. These typical risk factors of a twin pregnancy are greatly potentiated in the above mentioned setting.

**Conclusion:**

A twin pregnancy in the presence of a uterine malformation is rare and difficult to manage. These rare cases must be collected and reported in order to work out algorithms of monitoring and therapy as well as issue appropriate recommendations for their management.

## Background

Pregnancy and delivery in women with Müllerian anomalies are associated with numerous complications, including preterm labor and the need for greater attention to achieve successful parturition. The prevalence of congenital uterine anomalies varies among different populations: it is 5.5% in the general population, 8.0% in women with infertility, and 13.3% in women with a history of abortions. It peaks at 24.5% among patients with a history of abortions as well as infertility [1].

**Fig. 1:**
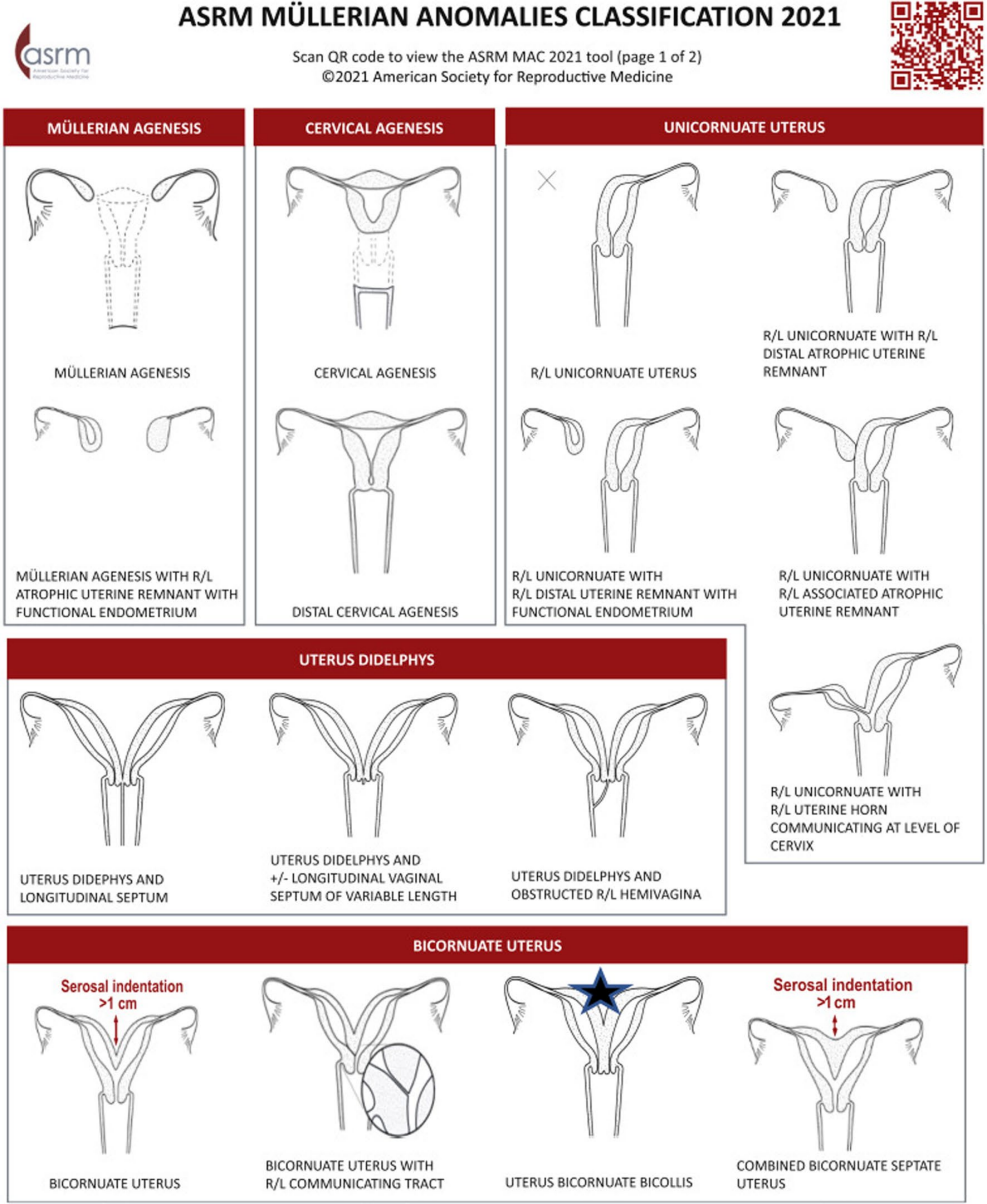
Printable version of the Müllerian Anomalies Classification Table by Pfeifer *et al*. The Müllerian anomaly described in this review is marked with a star [6]

The presence of a uterine anomaly is known to be associated with adverse pregnancy outcomes, including a higher risk of spontaneous abortion, preterm labor, cesarean delivery due to breech presentation, and reduced live births when compared to a normal uterus [2]. Nevertheless, the frequency of these outcomes varies across different types of uterine anomalies. Several classifications have been proposed, such as the vagina cervix uterus adnex-associated malformation (VCUAM) classification [3], the classification of the American Society of Reproductive Medicine (ASRM) [4] or the European Society of Human Reproduction and Embryology (ESHRE) and the European Society of Gynaecological Endoscopy (ESGE) [5]. A classification by the ASRM was established in a revision of the American Fertility Society (AFS) classification from 1988 (Fig. 1) [6].

The current prevalence of congenital uterine anomalies is a mere 5.5–6.7% in the general population, 7.3% in sterile women, and approximately 16.7% in women with recurrent miscarriage [1, 7]. The majority of bicornuate uteruses are asymptomatic and typically detected during a routine evaluation of the patient. In cases of more severe symptoms, such as primary amenorrhea, hematocolpos, pelvic pain, dyspareunia or obstacle during vaginal penetration, the condition is diagnosed early [7]. Otherwise, a uterine malformation is first diagnosed during obstetric complications, including spontaneous abortions, intrauterine growth restriction, preterm rupture of membranes, preterm labor, or malpresentation of the infant [8].

A bicornuate uterus is a rare congenital uterine malformation. A mere 16 cases of twin pregnancy associated with a bicornuate unicollis uterus have been reported worldwide, and most of these were delivered with a cesarean section [9, 10].

Pregnancies in the bicornuate uterus are typically regarded as a high-risk condition due to their association with adverse reproductive outcomes, including recurrent pregnancy loss, cervical insufficiency, low birthweight, preterm birth, malpresentation, cesarean delivery, and uterine rupture [2].

To our knowledge, this is the first report of a simultaneous pregnancy in each cavity of a bicornuate bicollis uterus and vaginal delivery of twins in the 28th week of pregnancy. The malformation was known prior to IVF and the embryo transfer was deliberately performed into one uterine horn to increase the likelihood of pregnancy.

## Case report

**Fig. 2:**
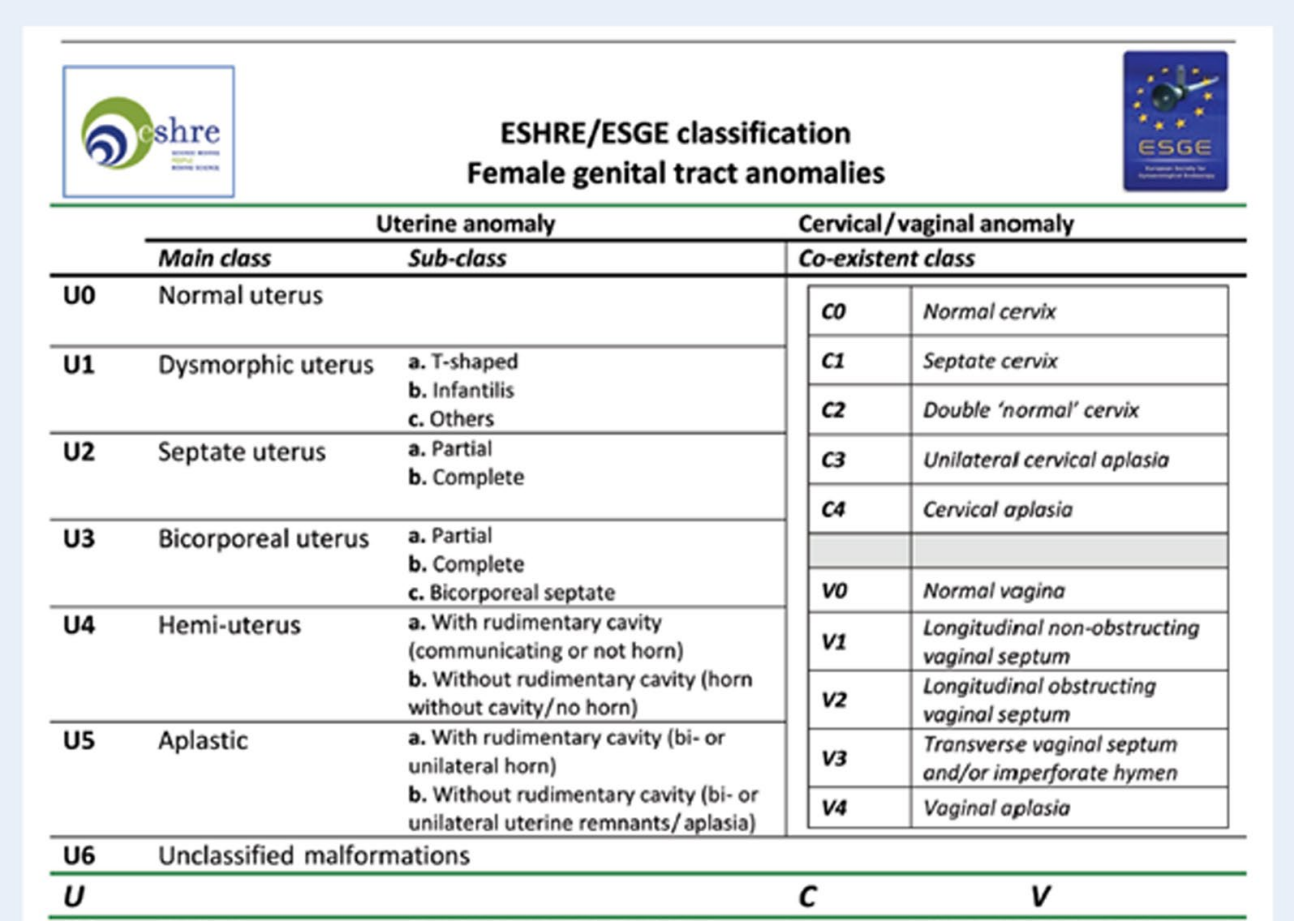
Vagina Cervix Uterus Adnex-associated Malformation classification by the European Society of Human Reproduction and Embryology/European Society of Gynaecological Endoscopy from Grimbizis *et al*. [5]

We report a 27-year-old European woman patient, gravida 1, para 1 at 27 + 2 weeks of gestation, with dichorionic diamniotic twin pregnancy and a bicornuate bicollis uterus. In 2021 the patient had undergone diagnostic laparoscopy with chromopertubation and hysteroscopy because she wished to have children. The procedure revealed a bicornuate bicollis uterus with two separate cervical canals arising from one portio. The uterus was seen on laparoscopy with a heart-shaped external contour (Fig. 1, marked with a star), V0 C1 U2 A0 M0 per the VCAUM classification (Fig. 2). After six unsuccessful inseminations and one frustrated intracytoplasmic sperm injection, parallel embryo transfer into one uterine horn each was performed in February 2023. This resulted in successful implantation of the embryos in one uterine horn each. The subsequent course of pregnancy history was initially physiological. The patient had an unremarkable first-trimester and second-trimester screening.

On 4 August 2023 the patient developed contractions and cervical shortening to 10 mm in the leading pregnancy. She received the first antenatal steroid prophylaxis consisting of 12 mg celestane under nifedipine tocolysis with supersaturation, and was then transferred to our level III perinatal center. On admission she continued to be symptomatic, with a prolapsed amniotic sac in the leading pregnancy and an otherwise unremarkable concordant dichorionic diamniotic twin pregnancy (first fetus: vertex presentation, estimated weight 916 g, left lateral placenta; second fetus: vertex presentation, estimated weight 1161 g left lateral placenta) with physiological amniotic fluid and fetal/fetomaternal Doppler findings. The tocolytic agent was switched to atosiban. The patient received antibiotic therapy with cefuroxime 1.5 g intravenously according to the regimen and a single oral dose of 1 g azithromycin for prolapse of the amniotic sac. She was admitted to the delivery room for further cardiotocography (CTG) monitoring. Detailed discussions were held with the patient about the possible mode of delivery. In view of all potential risks (including emergency cesarean section, cesarean section on the second fetus after spontaneous delivery of the first, atony, hysterectomy), we decided on a spontaneous delivery of the leading fetus with a possible wait-and-watch approach in regard of the second. The patient was informed verbally and in writing about the potential need for an emergency cesarean section and hysterectomy with bilateral salpingectomy. Our neonatologists and anesthesiologists also informed the patient about the subsequent procedures and the risks of preterm birth. Red blood cell concentrates were kept on call. With increasing labor activity, intravenous magnesium as neuroprotection was started per schedule. Nine hours later the patient presented with strong contractions, a sensation of pressure, and prolapse of the amniotic sac to the middle of the vagina. Tocolysis with atosiban was stopped, an amniotomy performed, and a male infant was born with the short-term support of an oxytocin infusion. With a birthweight of 1150 g (P 69), an Apgar score of 9/9/9, an arterial umbilical cord pH of 7.29, and base excess -7.70 mmol/l, the infant was transferred immediately to the neonatologists. The placenta followed spontaneously, the anesthesiology and surgery nursing team were sent away, and the patient remained well in the delivery room under further CTG monitoring. The ultrasound investigation revealed an empty right cavum while the left cervix appeared long and closed. In order to avoid uterine atony, the recommencement of tocolysis was initially postponed; the patient was currently labor free. After three hours her unstoppable contractions started again. With a pushing contraction she delivered the second infant: male, 1115 g (P 62), Apgar score 8/7/9, an arterial umbilical cord pH of 7.36, and base excess − 7.80 mmol/l. The infant was immediately transferred to the neonatologists. Three units of oxytocin as a short infusion were given and the placenta followed spontaneously. On ultrasound both uterine horns were empty and there was no birth injury or hemorrhage. The mother could be discharged after three days in good health.

Postnatally the first-born twin suffered from respiratory distress syndrome and was therefore given a surfactant twice by less invasive surfactant application (LISA) within the first 24 h after birth. The infant’s respiratory situation remained stable thereafter under non-invasive airway support. A patent ductus arteriosus (PDA) was treated successfully with ibuprofen. The clinical surveillance remained uneventful in regard of enteral feeding and there was no intraventricular bleeding.

The second twin also needed surfactant treatment for respiratory distress syndrome. A routine chest X-ray on the first day of the infant’s life suggested a sacculation of the esophagus, demanding endotracheal intubation for airway protection. Detailed imaging after transesophageal administration of a contrast agent revealed a remarkably large esophageal sacculation. The examination showed no fistulation towards the surrounding tissue or organs and no other signs of malformation. The newborn was extubated and a stable respiratory situation was maintained by non-invasive respiratory support. Next, endoscopic esophageal examination was performed to establish the need for any therapeutic intervention. A hemodynamically significant PDA was treated with ibuprofen and paracetamol. To date, a minimal residual PDA is being monitored closely and has revealed no hemodynamic irregularities. Similar to the twin brother, intracranial examinations in the second infant were uneventful and enteral feeding was established without complications.

**Fig. 3:**
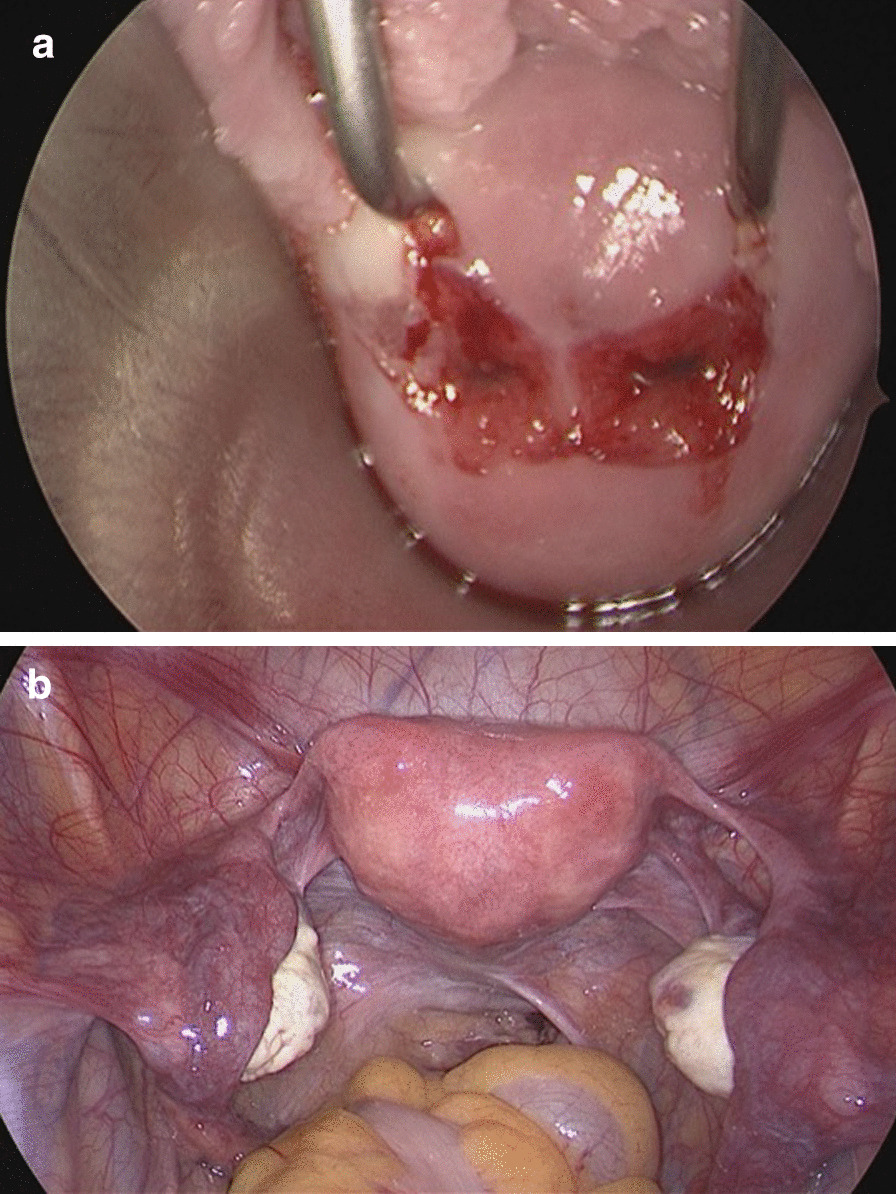
Intraoperative recording of hysteroscopy and laparoscopy, **a** intraoperative view of two separate cervical canals arising from one portio. **b** intraoperative view of a heart-shaped uterus and physiological adnexa

**Fig. 4:**
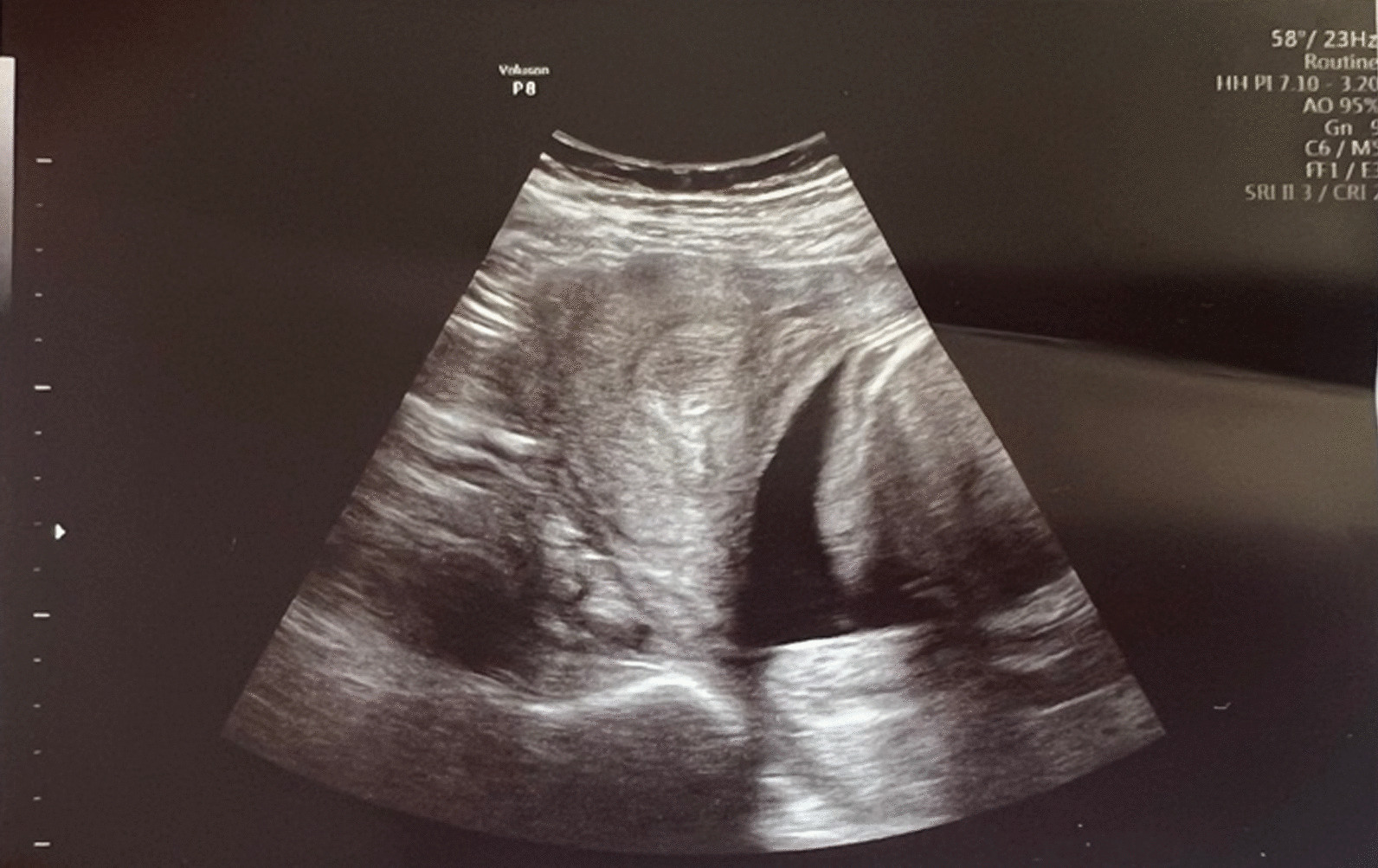
Ultrasound examination for control of contraction of the right uterine horn as well as exclusion of placental remnants after delivery of the first pregnancy

Figures 3 and 4 are intraoperative images of the uterine malformation on laparoscopy and an ultrasound performed during parturition.

**Fig. 5:**
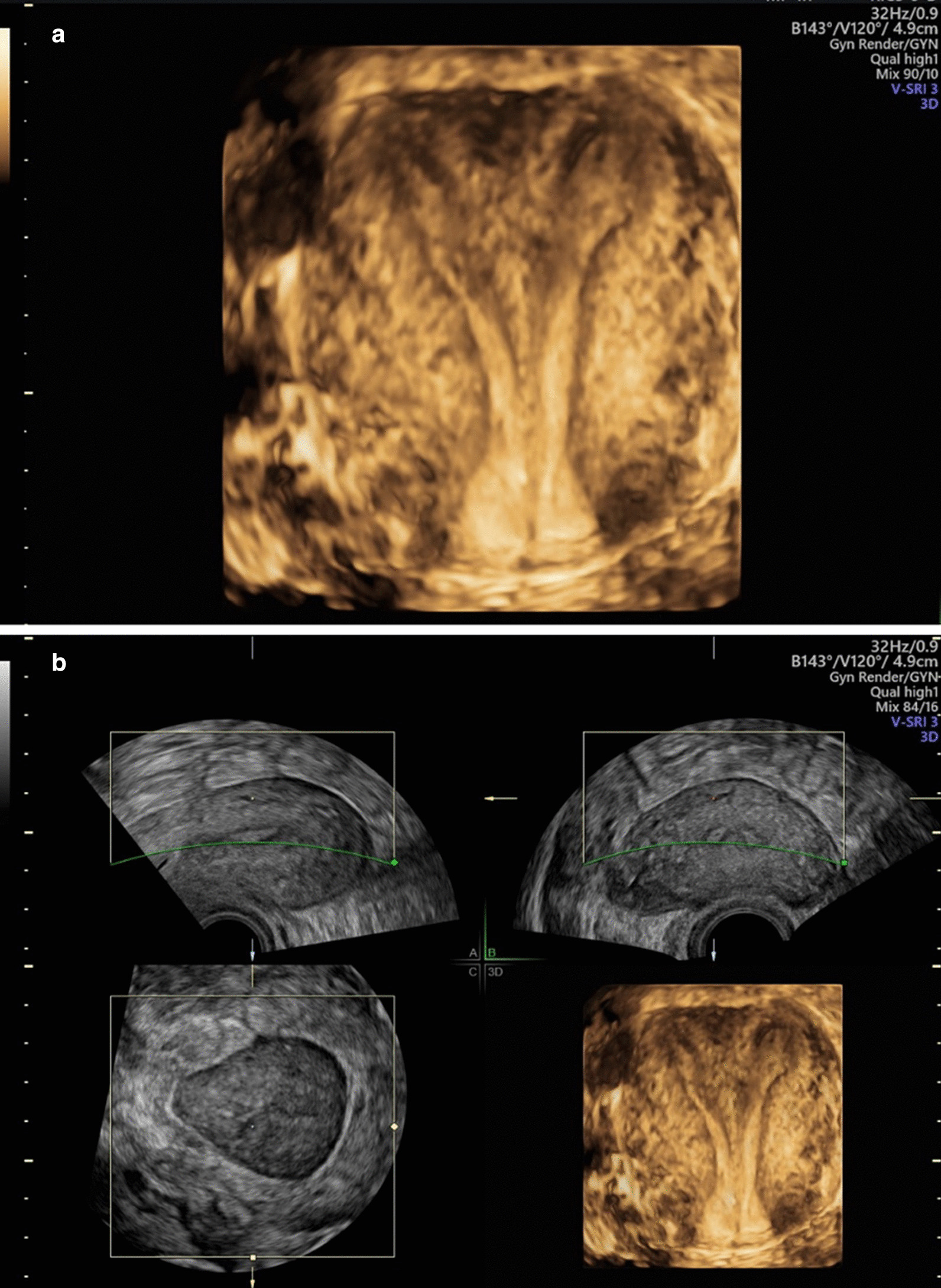
3D ultrasound at 8 weeks postpartum; **a** Bicornuate bicollis uterus (functional unicollis) in the transverse plane with both uterine cavities. **b** Bicornuate bicollis uterus (functional unicollis): cross-section through both uterine cavities, 3D ultrasound

The patient underwent a checkup, a 3D ultrasound investigation, and an MRI of the pelvis at eight weeks after delivery to confirm the diagnosis of a uterine malformation established previously by other colleagues. After a regular puerperium, the patient was in a good general condition. The speculum examination revealed an inconspicuous singular portio and the bimanual examination showed an enlarged anteflexed uterus with otherwise unremarkable findings. Three-dimensional ultrasound revealed a bicornuate uterus with a prominent septum extending to the portio (Fig. 5a, b). The ovaries as well as the kidneys appeared physiological on 2D ultrasound.

**Fig. 6:**
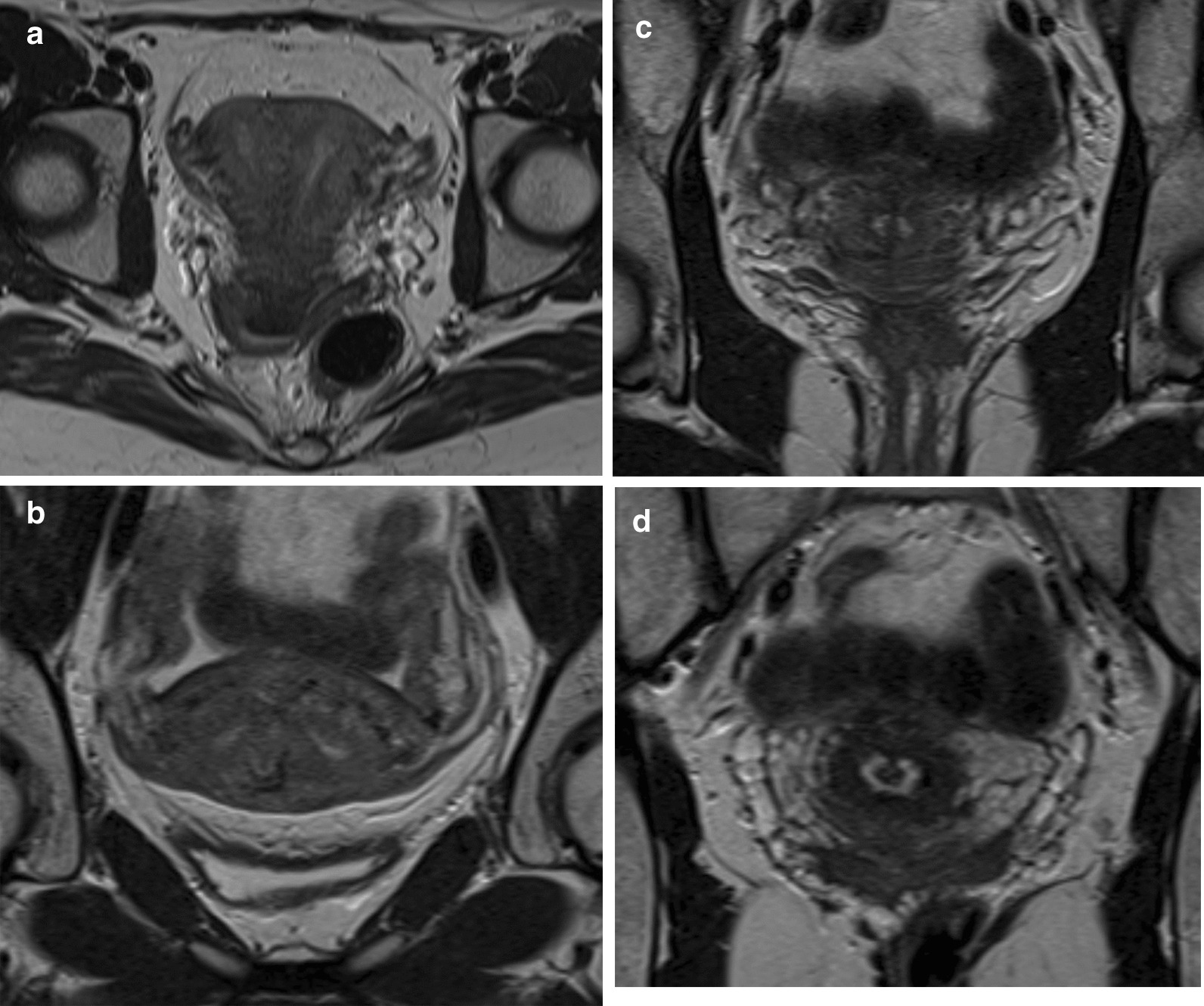
Magnetic resonance imaging at 8 weeks postpartum; **a** Bicornuate bicollis uterus (functional unicollis) in the transverse plane with both uterine cavities (transverse T2-weighted sequence, Avanto 1.5 T, Siemens Healthineers). **b** Bicornuate bicollis uterus (functional unicollis): cross-section through both uterine cavities near the fundus (coronary T2-weighted sequence, Avanto 1.5 T Siemens Healthineers). **c** Bicornuate bicollis uterus (functional unicollis): cross-section through both uterine cavities near the isthmus (coronary T2-weighted sequence, Avanto 1.5 T Siemens Healthineers). **d** Bicornuate bicollis uterus (functional unicollis): cross-section through the uterine cervix with the postpartum condition of a residual, partly strong cervical septum apically—possibly ruptured peripartum (coronary T2-weighted sequence, Avanto 1.5 T Siemens Healthineers)

A magnetic resonance imaging (MRI) of the pelvis confirmed our diagnosis of a functional bicornuate unicollis uterus with the postpartum condition of a residual, partly strong cervical septum apically, which possibly ruptured peripartum (Fig. 6a–d). Therefore, our diagnosis according to the VCUAM classification was V0 C + U2 A0 M0, a bicornuate uterus with a functional unicollis and a prominent septum.

## Discussion

The case described here is a rarity in terms of fertility medicine as well as obstetrics, with an exemplary successful outcome. Correct diagnosis of a uterine anomaly in the context of infertility, accurate conclusions, and IVF treatment after previously unsuccessful fertility attempts are important steps. Basic knowledge of uterine anomalies is a prerequisite for making correct decisions in each case. The next step is intensified pregnancy monitoring and knowledge of the existing risks. The final step is planning birth with due consideration to the risks involved.

A thorough understanding of embryology is essential when dealing with congenital anomalies of the uterus. The uterus, cervix, fallopian tubes and upper third of the vagina develop from the fused Müllerian ducts or paramesonephric ducts. Initially, fusion of the ducts is incomplete and a septum is present within the lumina. However, as time progresses the septum disappears and a single large cavity is formed [11, 12]. A bicornuate unicollis uterus is a congenital uterine malformation typically arising from incomplete fusion of the Müllerian ducts, leading to a varying degree of separation between the uterine cavities [12, 13]. Incomplete fusion of the Müllerian or paramesonephric ducts results in a bicornuate bicollis uterus, characterized by a double or single vagina, double cervices, and two single‐horned uteruses with partial fusion of their muscular walls. Renal anomalies as well as a vaginal septum may occur in these cases. In contrast, a uterus didelphys is seen in cases of complete lack of fusion of the Müllerian ducts, characterized in the majority of cases by two uterine cavities and two cervices with a longitudinal vaginal septum [12, 14].

Advanced investigation techniques such as MRI and 2D ultrasonography have made it easier to diagnose a malformed uterus [15]. Transvaginal 3D ultrasonography proved to be highly accurate in diagnosing and classifying congenital uterine anomalies, surpassing both hysteroscopy and MRI in its effectiveness. However, the management of uterine malformations is not a fixed procedure and varies according to the individual's clinical history [16, 17].

Uterine malformations may significantly impact pregnancy outcomes. Therefore, the misdiagnosis of such malformations must be avoided.

Women with a malformation of the uterus are confronted with poor chances of a normal pregnancy because their uterus does not offer the suitable physiological environment of a normal pear-shaped uterus. This condition may lead to obstetric complications such as infertility, ectopic pregnancy, and prematurity. Consequently, women with congenital uterine malformations have a low probability of conceiving naturally, causing the clinician and the patient to consider IVF. Surprisingly, after IVF/ Intracytoplasmic sperm injection (ICSI) treatment Kong *et al*. reported no difference in pregnancy rates between a bicornuate uterus and a normal uterus [18]. Chan *et al*. also reported that bicornuate uteruses do not reduce fertility. Women with a bicornuate uterus have a moderately increased risk of miscarriage, preterm birth and fetal malpresentation [19].

Currently, there is no established approach for the management of this type of pregnancy. Although vaginal delivery is not contraindicated, a cesarean section was performed in all 16 reported cases [20]. The reason for a cesarean section was an urgent indication for delivery, such as preeclampsia [8, 9] or the avoidance of obstetric complications such as malpresentation and uterine rupture [13, 21, 22]. A cesarean section was performed by two separate uterotomies in the lower uterine segment [9, 21, 22] or a bilateral vertical incision [14].

A preventive cerclage of the cervix may be considered, but is known to be associated with a poor reproductive outcome in uterine malformations [8]. Likewise, the National Institute for Health and Care Excellence (NICE) guidelines do not recommend a routine cervical cerclage to prevent spontaneous preterm birth in women with a twin or triplet pregnancy [23, 24].

Chemlal *et al*. reported a case of spontaneous twin pregnancy in a bicornuate unicollis uterus. The authors performed a preventive cervical cerclage with non-absorbable sutures at 12 weeks, and final delivery by cesarean section due to preeclampsia at 35 weeks [8].

Karunaratne *et al*. described a case of twin pregnancy in a patient with a bicornuate bicollis unterus and breech presentation of the fetus, delivered by primary cesarean section at 37 + 0 weeks. They also reviewed the literature and discussed several relevant points such as the feasibility of vaginal delivery versus cesarean section, the surgical approach in cesarean section, and delivery timing. They found that a cesarean section is preferentially used in cases of malpresentation, fetal distress and labor dystocia. The authors also give preference to longitudinal uterotomies. On the other hand, bilateral vertical incisions are recommended in small and externally fused lower uterine segments in order to avoid extension, injury to the septum, difficult repair and hemorrhage, and facilitate future pregnancies [14]. In the assumed presence of two functional uteruses and independent induction of labor, it would be advisable to adopt a two-stage approach. After the first delivery one would wait in order to prolong the second pregnancy and avoid a preterm birth. This approach has been successfully used in bicornuate bicollis uterus [25] as well as uterus didelphys [26].

**Fig. 7:**
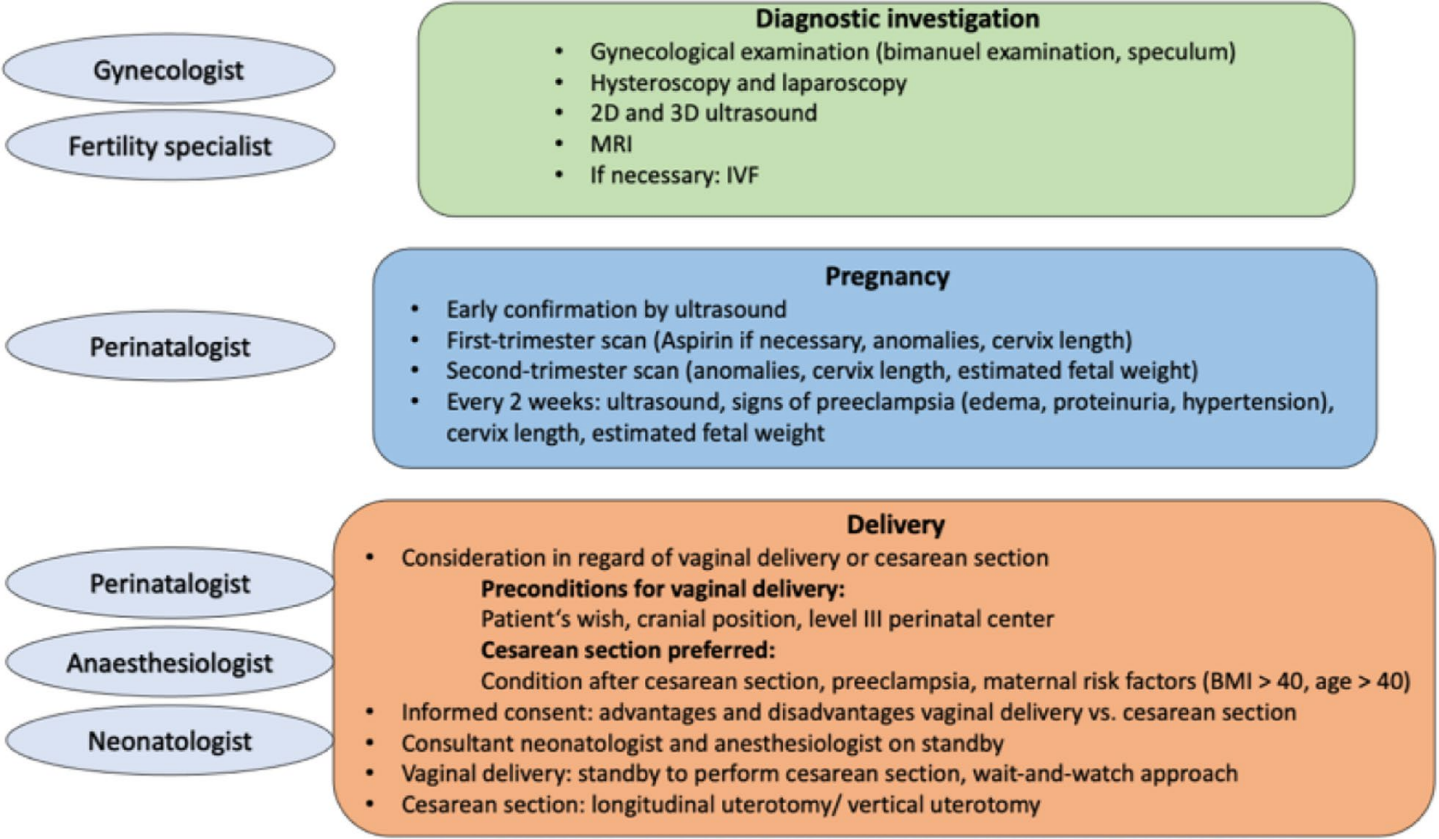
Comprehensive and intensive monitoring of patients with twin pregnancy in a bicornuate uterus

The optimal follow-up for twin pregnancies in patients with Müllerian fusion anomalies remains a subject of controversy, given the risks and the rarity of these cases. The treatment must be tailored to the individual case. Figure 7 summarizes our checklist for comprehensive and intensive monitoring of patients with twin pregnancy in a bicornuate uterus.

In our case, the postpartum 3D ultrasound and MRI led us to conclude that the correct classification of the uterine malformation was a bicornuate bicollis uterus, functional unicollis with a prominent septum extending to the portio. Our classification of the malformation—a functional unicollis rather than bicollis—would have led us to attempt spontaneous delivery. However, a wait-and-watch approach with interval delivery would not have been considered promising in view of the facts. Our current knowledge of the uterine anomaly offered the patient the option of performing a septum dissection later in order to raise the chances of spontaneous conception as well as reduce the risk of abortion and preterm birth.

## Conclusion

Uterine malformations must be detected early in order to prevent obstetric complications. Ultrasound, especially 3D ultrasound, and MRI play an important role in the detection of malformations. Twin pregnancy in a bicornuate uterus is very rare and bears a high risk of obstetric complication such as fetal growth restriction or preterm birth. Intensive monitoring is essential for successful management of these rare pregnancies. According to the guidelines, twin pregnancies should be monitored by serial ultrasound every two weeks in order to assess fetal growth and measure cervical length, and thus determine the risk of intrauterine growth restriction and premature delivery. However, we lack guidelines for monitoring these pregnancies and the mode of delivery.

Despite the rarity of this condition, a primary caesarean section should not be selected by default. The patient should be informed about the different delivery options and their risks. The mode of delivery should be determined individually. If spontaneous parturition is the goal, the clinician and the patient must be prepared for possible complications including emergency caesarean section and atony, and take interdisciplinary precautions that will avoid unnecessary delay in emergency situations.

These rare cases must be collected and reported in order to work out algorithms of monitoring and therapy, and provide suitable recommendations to the patients for an optimum outcome of pregnancy.

## Data Availability

The datasets analyzed for the current study are available from the corresponding author upon reasonable request.

## Abbreviations

AFS: American Fertility Society
ASRM: American Society of Reproductive Medicine
BMI: Body Mass Index
CTG: Cardiotocography
ESGE: European Society of Gynaecological Endoscopy
ESHRE: European Society of Human Reproduction and Embryology
IVF: *In vitro* fertilization
LISA: Less invasive surfactant application
MRI: Magnetic resonance imaging
NICE: National Institute for Health and Care Excellence
PDA: Patent ductus arteriosus
VCUAM: Vagina Cervix Uterus Adnex-associated Malformation
